# Integrated Health Solutions: Addressing the Co-occurrence of Otitis Media With Effusion and Early Childhood Caries in Preschool Children

**DOI:** 10.7759/cureus.68006

**Published:** 2024-08-28

**Authors:** Noor Dina Hashim, Chin Lee Lee, Farinawati Yazid

**Affiliations:** 1 Otorhinolaryngology-Head and Neck Surgery, Faculty of Medicine, Universiti Kebangsaan Malaysia, Kuala Lumpur, MYS; 2 Otolaryngology-Head and Neck Surgery, Hospital Sultanah Aminah, Johor Bahru, MYS; 3 Dentistry/Pediatric, Faculty of Dentistry, Universiti Kebangsaan Malaysia, Kuala Lumpur, MYS

**Keywords:** hearing loss, pediatric dental, speech delay, dental caries, otitis media with effusion

## Abstract

*Introduction:* Otitis media with effusion (OME) is a common middle ear condition, which frequently affects children and can lead to hearing impairment, speech delays, and developmental issues. Early childhood caries (ECC) is another prevalent pediatric condition defined by the presence of decayed, missing, or filled tooth surfaces in children under six years old.

*Objectives*: This study aims to determine the prevalence of OME and ECC, the association between these two conditions, and the risk and severity of ECC among preschool children with OME.

*Methodology*: A prospective cross-sectional study was conducted at a single tertiary center. A total of 206 preschool children aged six and below with hearing impairment or speech delay were recruited. They were grouped as follows: with OME (*n *= 129) and without OME (*n* = 77). Demographic and socioeconomic data were obtained, followed by otoscopy, anterior rhinoscopy, and tympanometric assessment. The subjects were further stratified into having ECC or not by conducting a dental examination for caries detection, whose findings were documented using Dental Charting and Caries Risk Assessment (CRA). Tympanometric width (TW) was also analyzed to measure its association with tympanic membrane appearance.

*Results:* 51.5% (n=106) of children with OME were found to have ECC. They predominantly had mild ECC (49.1%), and most (51%) were at moderate risk for caries based on the CRA (*P* < 0.001). A significant association between OME and ECC was observed (p < 0.001). Tympanometry results documented a strong correlation between TW greater than 200 daPa and abnormal tympanic membrane findings in OME (*P *< 0.001).

*Conclusions: *The association between OME and ECC in preschool children necessitates integrated healthcare approaches for early detection and management. Reliable diagnostic tools, such as tympanic width measurement for OME and CRA for ECC, are crucial in addressing these health issues. Early intervention and comprehensive care can mitigate the risks and improve health outcomes for affected children

## Introduction

Otitis media with effusion (OME) is a stealthy intruder in the world of childhood health, often creeping in unnoticed due to its lack of acute symptoms. This middle ear disease, the presence of mucus or fluid collection in the middle ear without any signs of acute infection, frequently eludes timely diagnosis, risking young children with subtle yet impactful symptoms such as recurrent ear discomfort, hearing impairment, delayed speech, and poor academic performance [1]. The prevalence of OME is staggering, with up to 90% of children experiencing it before school age, particularly between six months and four years old [2]. Many episodes resolve spontaneously but about 30%-40% may have recurrent OME. In Malaysia, the incidence of OME among children has risen from 13.8% to 18.3% over a decade, reflecting a growing concern [3,4]. Diagnosing OME typically involves pneumatic otoscopy, tympanometry, and pure tone audiometry. An active observation for 3 months is mandatory for newly diagnosed OME before surgical intervention and a short-term (<6 weeks) intranasal steroid can be offered for OME with concurrent allergic rhinitis (AR) and adenoid hypertrophy in children more than two years old. Surgical intervention should be considered after three months of persistent OME with conductive hearing loss >25 dB and/or structural changes to the tympanic membrane or middle ear despite optimum medical treatment.

Equally troubling is the prevalence of early childhood caries (ECC), a dental condition that affects children under six, characterized by decayed, missing, or filled teeth [5]. Nearly 40% of preschoolers in Los Angeles County suffer from ECC, a stark contrast to the 28% nationwide. In Malaysia, the prevalence among six-year-olds remains alarmingly high, declining only slightly from 80.9% in 1997 to 74.5% in 2007, far short of the WHO's goal of 50% caries-free children [6,7]. The risk factors for ECC are multifactorial, ranging from frequent consumption of sugary foods and bottle feeding to socioeconomic status. Untreated, ECC can lead to poor dentition, learning disabilities, and increased treatment costs, and it also serves as a precursor to permanent tooth caries.

Recent studies have uncovered a significant link between otitis media infections and ECC [7,8]. Sumer M et al reported nearly 30,000 children found a strong association between middle ear infections in the first year of life and subsequent ECC [9]. *Streptococcus mutans*, a common culprit in dental caries, has been found in higher concentrations in children with otitis media aged 5 and less. Bottle-fed children are at a heightened risk for both OME and ECC [8]. Intriguingly, a small-scale study in Istanbul identified *Fusobacterium nucleatum* in the oral cavity, nasopharynx, and middle ear secretions of children with OME and ECC [10], suggesting a microbial connection.

Despite the alarming statistics and potential for long-term impact, data on the prevalence of OME and ECC in preschool children remains scarce. This research aims to fill that gap by exploring the possible association between OME and ECC in young children and identifying reliable assessment methods to aid in the early diagnosis and management of these conditions. Ensuring good hearing and oral health during these formative years is crucial for children's speech, language development, and overall well-being.

## Materials and methods

Study design

This study is a prospective cross-sectional study conducted at the Otorhinolaryngology Clinic and Dental Clinic of a tertiary referral center. The ethical approval was obtained from the Ethics Committee of Universiti Kebangsaan Malaysia (PPI/111/8/JEP-2018-277). The primary objective was to investigate the prevalence and correlation of OME and ECC among Malaysian children aged six years or younger.

Sampling

A sample size of 206 was calculated using the Power and Sample Size program, considering a 10% dropout rate, to compare means between groups. Subjects were recruited based on specific inclusion criteria: healthy children aged six or younger with hearing impairment or speech delay. Children older than six, those without hearing or speech issues, uncooperative children, and those with acute ear infections were excluded. The study procedures were explained to the patients and their guardians, and informed consent was obtained.

Tools and outcome measures

Comprehensive demographic data including age, gender, ethnic group, medical history, and socioeconomic data were collected. Each child underwent an otoscopy examination followed by tympanometry to evaluate the tympanic membrane and middle ear compliance, and to measure the tympanometric width (TW) at 50% of its static acoustic admittance value in daPa. TWs exceeding the upper cut-off values (235 daPa for infants and 200 daPa for school-age children) are linked to OME according to the ASHA practice policy [11]. Dental charting was utilized to record the presence of caries, missing teeth, or filled tooth surfaces in primary teeth following WHO guidelines for epidemic studies [12]. In children under three years old, any indication of smooth-surface caries suggests severe ECC. Between ages three and five, severe ECC is defined as one or more cavitated, missing (due to caries), or filled smooth surfaces in primary maxillary front teeth, or a decayed, missing, or filled score of ≥4 (age 3), ≥5 (age 4), or ≥6 (age 5) surfaces according to the American Academy of Pediatric Dentistry [13]. Caries Risk Assessment (CRA) involves evaluating six factors to determine the overall risk for ECC. These factors include whether the mother or primary caregiver has active caries, if the child consumes more than three sugar-containing snacks or beverages between meals daily if the child is bottle-fed with sugary liquids in bed, if the child brushes their teeth daily with fluoridated toothpaste, and if the child has more than one decayed, missing, or filled tooth surface. Based on these factors, the assessment categorizes dental caries risk as low (2 or fewer risk factors), moderate (3-4 risk factors), or high risk (5-6 risk factors) [13].

Statistical analysis

The data were processed and analyzed using the IBM SPSS Statistics for Windows, Version 26.0 (IBM Corp., Armonk, NY). Descriptive analysis included frequency calculations for all categorical variables with 95% confidence intervals. Inferential analysis was conducted using the chi-square test to assess the association between categorical variables, such as the presence of OME and ECC. All subjects were examined by a single examiner to ensure consistency. Calibration was carried out by benchmark examiners, including an otorhinolaryngologist and a pediatric dental specialist, to maintain intra-examiner consistency.

## Results

In this study, we included 206 preschool children aged six and below, mostly boys (*n* = 126) and 80 girls. Among these children, 62.6% (129) were diagnosed with OME and 71.4% (147) had ECC (Table 1).

**Table 1:** Distribution of children with or without OME and ECC. OME, otitis media with effusion; ECC, early childhood caries

ECC					*P*-value
OME		No	Yes	Total	
	No	36 (17.5%)	41 (19.9%)	77	
	Yes	23 (11.10%)	106 (51.1%)	129	<0.001
Total		59	147	206	

In terms of ECC severity, 49.1% of children with both OME and ECC had mild ECC, 30.2% had moderate ECC, and 20.8% had severe ECC as documented using dental charting. This severity was significantly associated with OME (*P* < 0.001). CRA indicated that 51% of children with both conditions had a moderate risk of caries, also showing a significant association (*P* < 0.001), 51.5% who had OME also had ECC, with boys being more affected (54.3%) compared to 27.9% girls. The majority of affected children were two years old or younger (Table 2).

**Table 2:** Demographics of children with OME. OME, otitis media with effusion

OME group (*n* = 129)	OME with ECC (*n* = 106)	OME without ECC (*n* = 23)
Gender		
Male (%)	70 (54.3%)	6 (4.7%)
Female (%)	36 (27.9%)	17 (13.1%)
Age (years)		
2 and below	38 (29.5%)	8 (6.2%)
3	32 (24.8%)	6 (4.7%)
4	16 (12.4%)	6 (4.7%)
5	10 (7.6%)	1 (0.8%)
6	10 (7.6%)	2 (1.6%)

**Figure 1:**
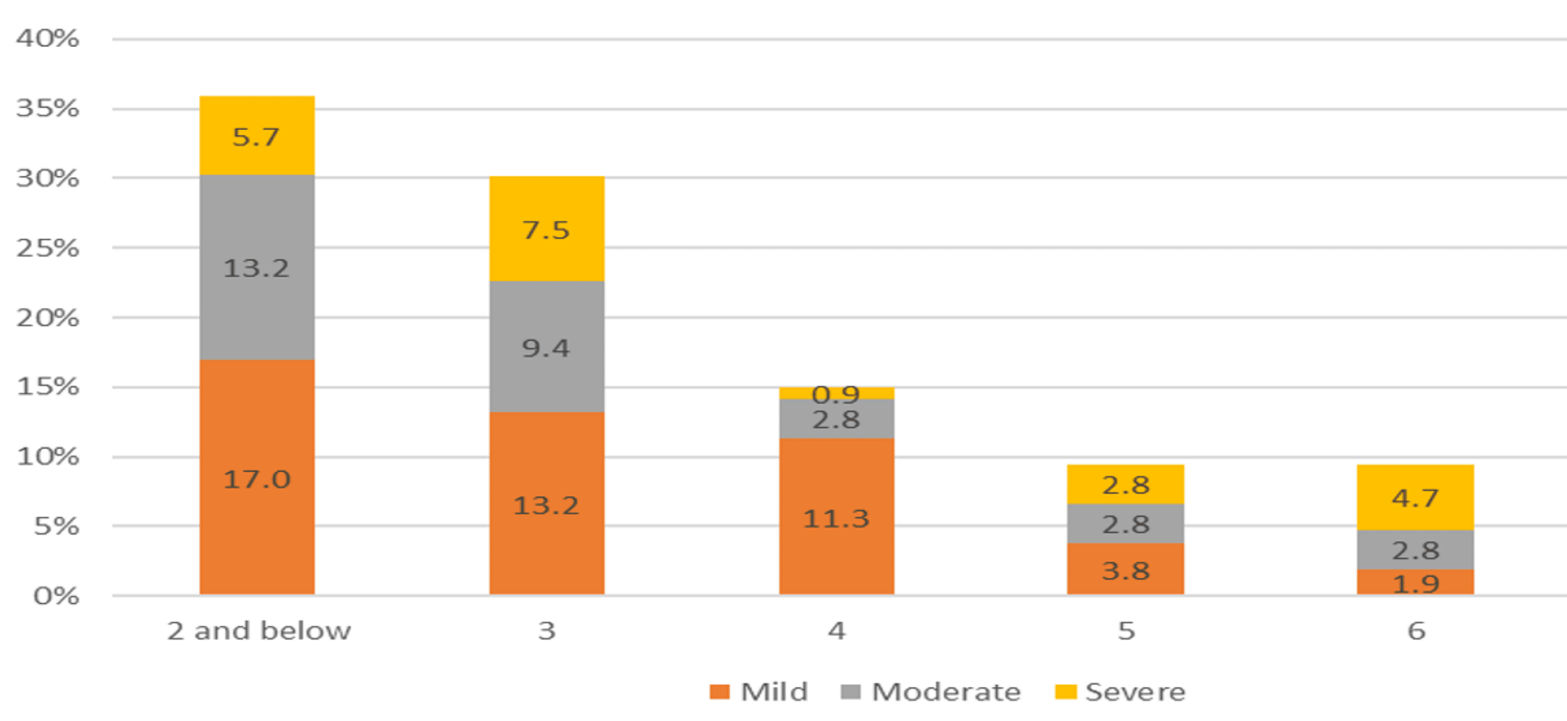
Severity of ECC according to different age groups among children with OME. OME, otitis media with effusion; ECC, early childhood caries

Using dental charting, nearly half (49.1%) of the children had mild ECC, with 30.2% experiencing moderate ECC and 20.8% presenting severe ECC. Children aged two and younger were notably more susceptible to caries, with 35.9% affected, predominantly with mild ECC (17.0%). Among three-year-olds, the prevalence of severe early childhood caries (ECC) was highest at 7.5% (Figure 1).

CRA indicated that 51% of these children had a moderate risk, 33% a high risk, and 16% a low risk of developing ECC. Importantly, a significant association between OME and ECC was established (*P *< 0.001), underscoring the heightened dental health challenges faced by children with OME and emphasizing the necessity for targeted preventive dental care interventions in this vulnerable population. 

TW was measured after conducting tympanometry tests. Results showed that 125 children with retracted tympanic membranes had TWs greater than 200 daPa, a statistically significant correlation (*P* < 0.001) (Table 3).

**Table 3:** Association between otoscopic finding and tympanometry width

		Tympanic Membrane		Total (n)	p- value
		Dull / Retracted / Bubble sign	Normal		
Tympanometric Width (daPa)	≤200	2	76	78	
	>200	125	3	128	<0.001
Total (n)		127	79	206	

Additionally, TWs over 200 daPa were observed in 128 children with a type B tympanogram, indicating OME (*P *< 0.001) (Table 4).

**Table 4:** Association between tympanogram and tympanometric width.

		Tympanometric width		Total	*P*-value
		≤200	>200		
Tympanogram	Type A (*n *= 68)	68	0	68	
	Type B (*n *= 129)	1	128	129	<0.001
	Type C (*n *= 9)	9	0	9	
Total		78	128	206	

While many children with both OME and ECC were in nursery care (63 children), there was no significant link between daily care settings and the incidence of these conditions. However, 68% of the subjects with both OME and ECC had underlying conditions such as AR and eczema, showing a significant correlation (*P* < 0.001).

## Discussion

OME and ECC are prevalent conditions that significantly impact the health and development of preschool-age children worldwide. Our study found a notable association between these two conditions, emphasizing the need for early diagnosis and integrated management strategies. OME and ECC are prevalent conditions that significantly impact the health and development of preschool-age children worldwide. OME, characterized by the accumulation of fluid in the middle ear without acute infection, often presents with subtle symptoms such as hearing difficulties, delayed language acquisition, and behavioral issues [10]. A diagnosis of OME is critical to preventing sequelae or complications that may lead to permanent or more debilitating conditions. Clinical diagnosis typically involves otoscopy, which can reveal a dull, retracted tympanic membrane and fluid levels or bubbles behind the membrane. In our study cohort, comprising children diagnosed with OME, we found that 61.7% exhibited clinical signs such as a retracted tympanic membrane or fluid levels on otoscopy. This underscores the high prevalence and clinical relevance of OME in young children, highlighting the importance of early detection and management to prevent potential complications like chronic effusion, conductive hearing loss, or even more severe sequelae such as cholesteatoma or intracranial infections [11].

In our study, we observed a noteworthy association between OME and ECC, with 51.5% of children affected by both conditions. This association was particularly pronounced among children aged two and younger, suggesting a critical period where these conditions coincide. This age group is vulnerable due to factors such as the horizontal orientation and wider diameter of the Eustachian tube, facilitating bacterial migration from the nasopharynx to the middle ear [9,10]. This aligns with the study by Goulioumis et al. reported that young children are at a heightened risk for concurrent OME and ECC due to anatomical and immunological factors, such as the horizontal orientation of the Eustachian tube and an underdeveloped immune system [13]. Additionally, the poorly developed immune defense and the presence of adenoids and lymphoid follicles in the throat further predispose young children to both OME and ECC, as highlighted in the work by Mashat et al. [14]. Several studies have explored the microbial link between OME and ECC, supporting the hypothesis that similar bacterial pathogens are involved in both conditions. Brook demonstrated the presence of anaerobic bacteria and anaerobic flora isolated from children with otitis media suggesting a shared pathophysiological mechanism [15]. Our study supports these findings, indicating that children with OME are at an increased risk for developing ECC, potentially due to the shared colonization by these pathogens [9].

The high prevalence of OME and ECC in our study cohort underscores the importance of early and accurate diagnosis. Otoscopy and tympanometry are essential diagnostic tools for detecting OME, with TW proving to be a particularly useful indicator of middle ear effusion. TW of greater than 200 daPa with low peak admittance had a sensitivity of 83% and a specificity of 87%, which is superior to the common practice of using tympanometry, as reported by Rosenfeld et al. [16]. Abnormal tympanic membranes in our subjects have shown a strong association with a TW > 200 daPa. This finding advocates the use of TW in diagnosing OME, especially in doubtful cases or in uncooperative children in whom clinical examination is a strenuous task [16,17].

Parallel to OME, ECC represents a significant oral health challenge affecting infants and young children. ECC manifests as localized tooth decay due to bacterial fermentation of dietary carbohydrates, leading to acid production that demineralizes dental hard tissues [18]. Diagnosis involves both visual and clinical examination, with ECC severity categorized based on clinical appearance. Risk indicators that indirectly influence the course of the disease process include socioeconomic status, lifestyle habits, income, education, knowledge, and oral health awareness attitudes [18,19].

CRA emerged as a pivotal tool for stratifying ECC risk among children with OME. This systematic evaluation and identification of risk factors focuses more on lifestyle risks that may contribute to the eruption of caries and/or the progression of an existing ECC [12,20]. In this cohort, more than half of the children assessed were at moderate risk for developing ECC, and the majority of these children already had mild ECC when screened through dental charting. Alarmingly, severe ECC cases were already prevalent at the time of assessment, potentially impacting speech development and overall oral health. This underscores the urgency of implementing preventive measures early in childhood to mitigate the risk of dental decay and associated developmental consequences by adopting CRA instead of just dealing with the symptoms of the disease [20,21].

OME and ECC represent intertwined childhood health challenges that require integrated management approaches. Early diagnosis, comprehensive risk assessment through tools like CRA, and targeted interventions are crucial to mitigating the long-term impacts on speech development, hearing, and oral health [22,23]. A study by Norhafizah et al. found a high prevalence of AR among children with OME, suggesting that underlying medical conditions may predispose children to both OME and ECC [24]. This supports our findings and highlights the importance of considering comorbid conditions in the management of these children. Kim et al. demonstrated that socioeconomic factors, dietary habits, and oral hygiene practices are significant determinants of ECC prevalence among children with OME [25]. These findings underscore the need for comprehensive risk assessments that consider a range of environmental and lifestyle factors. Multidisciplinary collaboration among healthcare providers, educators, and parents is essential to improve awareness, implement effective prevention strategies, and enhance overall outcomes for children affected by these conditions.

The strengths of the study include good calibration and consistency of the dental examination, the use of validated diagnostic tools such as otoscopy, tympanometry, and dental charting and sufficient sample size. However, this study may face several limitations. Firstly, the sample was drawn from a single tertiary referral center, potentially limiting the generalizability of findings to broader populations. Selection bias could be present as the study focused on children with speech or hearing issues, potentially overlooking those who are asymptomatic or have milder forms of OME or ECC. Additionally, confounding factors such as socioeconomic status, dietary habits, and oral hygiene practices were not fully controlled, possibly influencing the observed associations. The cross-sectional design of this study limits the ability to establish causality between OME and ECC, highlighting the need for longitudinal studies. Addressing these limitations could strengthen future research efforts to better understand the complex relationship between OME and ECC in preschool-age children.

## Conclusions

Our study highlights the significant association between OME and ECC in young children and underscores the need for early diagnosis and integrated management strategies. Addressing both conditions holistically is crucial for mitigating their long-term effects on hearing, speech development, and oral health. By implementing comprehensive risk assessments and early interventions, healthcare providers can effectively reduce the societal and developmental burdens associated with these prevalent childhood conditions.
